# Supplementary material for: Empirical methods for controlling false positives and estimating confidence in ChIP-Seq peaks
Source: BMC Bioinformatics. 2008 Dec 5;9:523. doi: 10.1186/1471-2105-9-523 (PMC2628906; doi:10.1186/1471-2105-9-523)
Supplement: Additional File 1 — A variety of html documents from the USeq web site detailing the available applications, their best usage, output file type descriptions, command line menus, etc. [file 1471-2105-9-523-S1.zip › USeqUserGuides/JavaDoc.zip!/JavaDoc/util/bio/parsers/BaseCount.html]

BaseCount


|  |  |  |
| --- | --- | --- |
| **Overview** \| **Package** \| **Class** \| **Use** \| **Tree** \| **Deprecated** \| **Index** \| **Help** | |  |
| **PREV CLASS**   **NEXT CLASS** | **FRAMES**    **NO FRAMES**     **All Classes** |  |
| SUMMARY: NESTED \| FIELD \| CONSTR \| METHOD | DETAIL: FIELD \| CONSTR \| METHOD |  |


---


## util.bio.parsers Class BaseCount

```
java.lang.Object
  util.bio.parsers.BaseCount
```

---

``` public class BaseCount extends java.lang.Object ```

---

**Constructor Summary**

`BaseCount(int position,
char base)`


| **Method Summary** | |
| --- | --- |
| `void` | `countBase(char b)`             Base must be lower case, if not gatc then n is incremented. |
| `int` | `getA()` |
| `char` | `getBase()` |
| `int` | `getC()` |
| `int` | `getDeletions()` |
| `int` | `getG()` |
| `java.util.ArrayList<java.lang.Integer>` | `getInsertionLengths()` |
| `int` | `getN()` |
| `int` | `getPosition()` |
| `int` | `getT()` |
| `void` | `setA(int a)` |
| `void` | `setBase(char base)` |
| `void` | `setC(int c)` |
| `void` | `setDeletions(int deletions)` |
| `void` | `setG(int g)` |
| `void` | `setInsertionLengths(java.util.ArrayList<java.lang.Integer> insertionLengths)` |
| `void` | `setN(int n)` |
| `void` | `setPosition(int position)` |
| `void` | `setT(int t)` |
| `java.lang.String` | `toString()` |

**Methods inherited from class java.lang.Object**

`equals, getClass, hashCode, notify, notifyAll, wait, wait, wait`

**Constructor Detail**

### BaseCount

```
public BaseCount(int position,
                 char base)
```


**Method Detail**

### countBase

```
public void countBase(char b)
```

:   Base must be lower case, if not gatc then n is incremented.

---


### toString

```
public java.lang.String toString()
```

:   **Overrides:**: `toString` in class `java.lang.Object`

---


### getA

```
public int getA()
```

---


### setA

```
public void setA(int a)
```

---


### getBase

```
public char getBase()
```

---


### setBase

```
public void setBase(char base)
```

---


### getC

```
public int getC()
```

---


### setC

```
public void setC(int c)
```

---


### getDeletions

```
public int getDeletions()
```

---


### setDeletions

```
public void setDeletions(int deletions)
```

---


### getG

```
public int getG()
```

---


### setG

```
public void setG(int g)
```

---


### getInsertionLengths

```
public java.util.ArrayList<java.lang.Integer> getInsertionLengths()
```

---


### setInsertionLengths

```
public void setInsertionLengths(java.util.ArrayList<java.lang.Integer> insertionLengths)
```

---


### getN

```
public int getN()
```

---


### setN

```
public void setN(int n)
```

---


### getPosition

```
public int getPosition()
```

---


### setPosition

```
public void setPosition(int position)
```

---


### getT

```
public int getT()
```

---


### setT

```
public void setT(int t)
```


---


|  |  |  |
| --- | --- | --- |
| **Overview** \| **Package** \| **Class** \| **Use** \| **Tree** \| **Deprecated** \| **Index** \| **Help** | |  |
| **PREV CLASS**   **NEXT CLASS** | **FRAMES**    **NO FRAMES**     **All Classes** |  |
| SUMMARY: NESTED \| FIELD \| CONSTR \| METHOD | DETAIL: FIELD \| CONSTR \| METHOD |  |


---
